# Comment on “Clinical Comparisons of Two Free Light Chain Assays to Immunofixation Electrophoresis for Detecting Monoclonal Gammopathy”

**DOI:** 10.1155/2015/742762

**Published:** 2015-03-12

**Authors:** Antony R. Parker, Oscar Berlanga, Stephen Harding

**Affiliations:** Binding Site Group Limited, 15 Calthorpe Road, Birmingham B15 1QT, UK

The recent publication by Kim et al. [1] compares the performance of two serum free light chain (sFLC) assays, the polyclonal antibody based Freelite and the monoclonal antibody based N-Latex-FLC. We welcome the opportunity to comment on the design of the study and interpretation of results.

Serum and urine electrophoresis can be used to identify monoclonal gammopathy (MG) patients with gross intact monoclonal immunoglobulin or free light chain production. However, electrophoresis assays are insensitive for the detection of patients with AL amyloidosis and nonsecretory multiple myeloma (NSMM). The introduction of the Freelite assay in 2001 [2] improved the sensitivity for detection of patients with monoclonal free light chain production. This improved sensitivity has resulted in the inclusion of Freelite in international guidelines [3–5] for screening, diagnosis, and monitoring of monoclonal gammopathies. Recently, assays utilising monoclonal antibodies for the measurement of serum free light chains have become available. The assays are calibrated to Freelite, but so far there is a paucity of data comparing the clinical performance of the assays.

Kim et al. analysed samples from 63 MG (*n* = 100 samples) and 57 non-MG (*n* = 57 samples) patients. Both kappa and lambda N-Latex-FLC are calibrated to the Freelite assays [6] and therefore it is not surprising that there is concordance with results in a normal population. However, we believe there are too few clinical samples from patients with light chain multiple myeloma (LCMM) (10/63) and AL amyloidosis (2/63) for Kim et al. to make a reliable clinical comparison.

There were 17 discordant results in this study (13 MG and 4 non-MG patients). It would have been informative if the authors had presented the performance of the assays in the different groups of monoclonal gammopathy patients, particularly in those with LCMM and AL amyloidosis. Specifically in LCMM populations previous studies with the monoclonal antibody based N-Latex-FLC test have failed to identify all patients [6–8]. By contrast in sixteen independent studies, including samples from 682 LCMM patients, an abnormal *κ*/*λ* sFLC ratio using the Freelite assay identified 100% samples (Table 1) [9–25]. One study [26] reported a LCMM patient missed by Freelite; however the sample was correctly identified when reanalysed using the same batch of reagent, indicating a previous analytical error (personal communication). To truly understand the concordance between the assays larger studies are required in clinically relevant populations including patients with AL amyloidosis, LCMM, and NSMM. In addition, there has only been a single study comparing the assays in patient with acute kidney injury [27].

4/57 non-MG patients had an abnormal *κ*/*λ* sFLC ratio using the Freelite assay but had normal ratios using the N-Latex-FLC assay. These patients had disorders (chronic kidney disease, chronic obstructive pulmonary disease, iron deficiency anaemia, and systemic lupus erythematosus) that have previously been reported to cause a perturbation of the *κ*/*λ* sFLC ratio due to poor renal function, inflammation, or immune stimulation [28–30]. In patients with renal impairment FLC removal becomes increasingly dependent on the reticuloendothelial system. Unlike renal clearance reticuloendothelial clearance is not influenced by size of the light chains [31]; therefore the production rate of kappa FLC (approximately 2x that of lambda) exerts an influence on the *κ*/*λ* FLC ratio. Whilst there have previously been reports highlighting the difference in the performance of the N-Latex-FLC assay in patients with impaired renal function, there has been no physiological explanation for this performance [32].

The quantitative assessment of free light chains by Freelite is an important laboratory test. An abnormal ratio can be used as part of an algorithm to risk stratifying monoclonal gammopathy of undetermined significance patients. Furthermore, a ratio of >100 with a monoclonal free light chain concentration >100 mg/L was recently included in the diagnostic algorithm for patients with multiple myeloma, and an abnormal ratio is useful in understanding the depth of response in patients during the course of their disease [33–35]. Given the reliance upon the numerical values we believe there is a strong requirement for better quantitative concordance between the assays, and clearly the role of Freelite in diagnosis, stratification, and response cannot be transferred to the N-Latex-FLC assay.

In summary, sample selection in this study limits interpretation but supports published data showing that differences exist between the polyclonal and monoclonal FLC assays.

## Figures and Tables

**Table 1:** Publications reporting detection of LCMM patients with Freelite.

Study (year)	Patient numbers	*κ*	*λ*	*κ*/*λ* ratio abnormal
Bradwell et al. 2003 [9]	224	123	101	100%
Drayson et al. 2009 [10]	223	NA	NA	100%
van Rhee et al. 2007 [11]	49	NA	NA	100%
Kraj et al. 2011 [12]	37	21	16	100%
Abraham et al. 2002 [13]	28	9	19	100%
Avet-Loiseau et al. 2011 [14, 15]	25	14	11	100%
Kang et al. 2005 [16]	23	14	9	100%
Nowrousian et al. 2005 [17]	17	NA	NA	100%
Hutchison et al. 2008 [18]	13	5	8	100%
Mösbauer et al. 2007 [19]	9	5	4	100%
Piehler et al. 2008 [20]	7	4	3	100%
Harding et al. 2009 [21]	7	4	3	100%
Lebovic et al. 2007 [22]	7	3	4	100%
Giarin et al. 2006 [23]	6	NA	NA	100%
Wolff et al. 2007 [24]	5	NA	NA	100%
Dogaru et al. 2011 [25]	2	0	2	100%
